# Metacognitive Change During Exposure and Metacognitive Therapy in Obsessive-Compulsive Disorder

**DOI:** 10.3389/fpsyt.2021.722782

**Published:** 2021-09-03

**Authors:** Jana Hansmeier, Anke Haberkamp, Julia A. Glombiewski, Cornelia Exner

**Affiliations:** ^1^Department of Psychology, University of Leipzig, Leipzig, Germany; ^2^Department of Psychology, University of Marburg, Marburg, Germany; ^3^Department of Psychology, University of Koblenz-Landau, Mainz, Germany

**Keywords:** exposure and response prevention, metacognitive therapy, stop signals, metacognitions, obsessive-compulsive disorder

## Abstract

Metacognitive therapy (MCT) has been shown to be a promising treatment approach for obsessive-compulsive disorder (OCD). The changeability of metacognitions by (metacognitive) treatment and its relevance to treatment outcome is, however, still unclear. The current study investigates, (1) if treatment with MCT or exposure and response prevention (ERP) in a randomized-controlled pilot trial (*n* = 24 patients with OCD) changes OCD-specific metacognitions of thought fusion beliefs, beliefs about rituals and stop signals, and (2) if these changes are relevant for the treatment outcome in terms of patient- and therapist-rated OCD symptoms. ANOVA with pretest, posttest and follow-up scores could show that all three metacognitions significantly decreased during both treatments. Regarding thought fusion beliefs, a significant interaction effect indicated a higher decrease after MCT than ERP treatment. In hierarchical regression analyses, changes in stop signals from pre- to post-treatment significantly predicted patient-rating OCD symptoms at post-treatment and follow-up at 3 months after treatment. These changes were even predictive of post-treatment outcome after controlling for general metacognitions and dysfunctional cognitive beliefs. These findings support the assumption that metacognitions can change during both treatments and that changes in stop signals might be relevant for the treatment outcome on the symptom level in OCD.

## Introduction

Metacognitive therapy (MCT) (1) has been suggested as one promising treatment approach for obsessive-compulsive disorder (OCD) (2). Pilot and single case studies found support for the efficacy for MCT for OCD [e.g., (3, 4)]. In the largest uncontrolled trial (5), 80% of OCD patients treated by MCT yielded a clinically significant change at follow-up. Also in a routine clinical service, MCT applied as a group treatment was found to be effective in OCD, with higher clinical response rates than a group cognitive behavior therapy (6). We recently reported results of a controlled pilot study showing that MCT was equally effective as the gold standard for treatment of OCD, exposure with response prevention (ERP) in reducing OCD symptoms (7). MCT required less treatment time than ERP in this study.

This raises the question of which mechanisms of change might be relevant for the treatment outcome in OCD with metacognitive approaches. Specifically, three obsessive-compulsive- (OC-) specific metacognitive beliefs are supposed to be modified in MCT for OCD: thought fusion beliefs about the meaning and power of intrusive thoughts (e.g., “Thinking about harming some will make me do it”), positive beliefs about the need to carry out rituals (e.g., “I need to perform my rituals otherwise I will never have peace of mind.”) and subjective stop signals for monitoring the actions (e.g., “An important signal when I can stop my rituals is when I feel calm”). Contrary to cognitive models of OCD [e.g., (8)] that focus on dysfunctional cognitive belief contents like perfectionism and inflated responsibility, MCT thereby aims to change thought *processes* instead of thought *contents*. Empirical evidence for the relevance of the OC-specific metacognitions for OCD comes from a wide range of studies [for a review, see Fisher (2)]: Cross-sectional studies showed that OC-specific metacognitions predict OCD symptoms, even when controlling for cognitive beliefs [e.g., (9)]. In addition to prospective studies (10), experimental studies [e.g., (11, 12)] support the causal role of metacognitions in OCD. In comparison studies [e.g., (13, 14)], individuals with OCD reported significantly more positive beliefs about rituals and stop signals than other (non-)clinical groups.

To date, three studies have investigated metacognitions as a potential mechanism of change in the treatment of OCD (15–17). All studies found a prediction of treatment outcome by a change in metacognitions, with two studies (15, 17) showing a better prediction by changes in metacognitions than by changes in cognitive beliefs in ERP. However, Solem et al. (17) and Kim et al. (16) investigated general and not OC-specific metacognitions. General metacognitions, such as positive beliefs about the usefulness of worry, are supposed to be relevant for a wide range of mental disorders rather than only OCD (18). In the study by Grotte et al. (15), only thought fusion beliefs and beliefs about rituals, but not stop signals were considered as mechanisms of change. However, stop signals have been found to be especially relevant for OCD symptoms [e.g., (9)]. In addition, the authors did not investigate whether MCT is especially beneficial in changing these beliefs, and they only used self-rated measures of OCD symptoms as the treatment outcome.

The present study aimed to investigate the changeability of OC-specific metacognitions by MCT and ERP, and its relevance to treatment outcome in OCD. To investigate the relevance of these metacognitions in comparison with other supposedly relevant mechanisms of change, general metacognitions and dysfunctional cognitive beliefs were also considered in the respective analyses. Specifically, the following hypotheses were examined: (1) both MCT and ERP can reduce OC-specific metacognitions, albeit with MCT achieving a higher decrease of dysfunctional metacognitive beliefs than ERP. (2) Changes in OC-specific metacognitive beliefs are relevant for the treatment outcome in OCD as measured with global OCD symptom scores. (3) Changes in OC-specific metacognitive beliefs are more relevant than changes in general metacognitions and dysfunctional cognitive beliefs for the treatment outcome. The treatment outcome of OCD symptom scores was measured by both self- and clinician-rated measures.

## Method

### Participants

The sample consisted of 24 German-speaking individuals with the main diagnosis of OCD according to DSM-IV. They represented a subsample of the completer sample (*n* = 28) of a pilot trial comparing ERP and MCT (7). Four patients of the completer sample of the pilot trial did not complete questionnaires measuring OC-specific metacognitions at posttreatment so that they were not considered in the current analyses. Inclusion criteria were: (a) diagnosis of OCD according to DSM-IV, and (b) 18–65 years of age. Exclusion criteria were as follows: (a) life time diagnosis of substance dependence, psychosis, neurological conditions, and (b) intellectual disability. The German version (19) of the Structured Clinical Interview (SCID) was administered to assess DSM-IV-TR current and lifetime disorders.

### Procedures

The data of the current study is part of a pilot trial comparing ERP and MCT (7), which was registered with ClinicalTrials.gov (NCT01483339). The study was approved by the Institutional Review Boards of both universities, the University of Marburg and the University of Leipzig. Participants were recruited from consecutive referrals to the universities' outpatient clinics. After screening of eligibility and informed consent, patients were randomly assigned to ERP or MCT. Random assignment was stratified by a diagnosis of comorbid depression. During the follow-up period of 3 months, three short telephone booster sessions following a fixed protocol took place.

### Treatment Conditions

The MCT protocol as proposed by Wells (1) was slightly adjusted for the study. The original treatment schedule of ten treatment sessions was extended to 14 sessions to allow for adaptions to individual needs of patients. Verbal methods (e.g., socratic questioning about evidence, reframing advantages), detached mindfulness and behavioral experiments (e.g., ritual postponement) were applied during MCT to change metacognitions. According to the ERP protocol (20), prolonged ERPs were implemented in therapist-guided in-session and between-session self-exposures after preparing and planning the individual treatment (e.g., psychoeducation about habituation, hierarchy of anxiety-provoking situations). An overview of the contents of both treatment protocols is presented in Glombiewski et al. (7). In both conditions, 14 individual weekly sessions were offered. In ERP, one session could last longer than 50 min depending on the individual length of exposure. Thereby, the number of treatment hours (á 50 min) was significantly higher in the ERP than in the MCT condition (see Table 1).

**Table 1:** Demographic and clinical characteristics of participants.

**Variable^a^**	**Total (***n*** = 24)**	**ERP (***n*** = 12)**	**MCT (***n*** = 12)**	**Statistic**	***P*** **^b^**
**Demographic**					
Age, y	30.5 ± 10.4	28.7 ± 9.4	32.3 ± 11.4	*t*_(22)_ = −0.862	0.398
Education^c^, y	14.6 ± 3.1	15.0 ± 3.7	14.3 ± 2.6	*t*_(20)_ = 0.531	0.601
Gender, no. (%) female	15 (63)	9 (75)	6 (50)	$\chi_{\left(1\right)}^{2}$ = 1.600	0.206
**Clinical**					
Duration of disorder, y	8.1 ± 7.9	8.6 ± 10.1	7.6 ± 5.0	*t*_(21)_ = −0.319	0.753
Any current co-morbid disorder^d^, no. (%)	10 (42)	5 (42)	5 (42)	$\chi_{\left(1\right)}^{2}$ = 0.000	1.000
Current depression^d^, no. (%)	6 (25)	3 (25)	3 (25)	$\chi_{\left(1\right)}^{2}$ = 0.000	1.000
Y-BOCS, Total, pre	23.9 ± 6.4	24.1 ± 5.6	23.5 ± 7.3	*t*_(22)_ = −0.220	0.828
BDI-II, Total, pre	20.0 ± 10.3	20.9 ± 9.8	19.2 ± 11.2	*t*_(22)_ = 0.397	0.695
**Metacognitions**					
TAF scale, pre	1.5 ± 0.8	1.4 ± 0.9	1.7 ± 0.7	*t*_(22)_ = −0.943	0.356
BARI, pre	2.5 ± 0.6	2.3 ± 0.5	2.6 ± 0.7	*t*_(22)_ = −1.535	0.139
SSQ, pre	2.5 ± 0.7	2.6 ± 0.7	2.5 ± 0.7	*t*_(22)_ = 0.418	0.680
**Treatment**					
Treatment sessions, no.	13.3 ± 1.4	13.1 ± 1.6	13.5 ± 1.2	*t*_(22)_ = −0.739	0.467
Treatment hours^e^, no.	18.2 ± 6.7	22.9 ± 6.7	13.5 ± 1.2	*t*_(11.7)_ = 4.782	**<0.001**

*ERP, Exposure with response prevention; MCT, Metacognitive therapy; Y-BOCS, Yale-Brown Obsessive-Compulsive Scale; BDI, Beck Depression Inventory; TAF scale, Thought-Action Fusion scale; BARI, Beliefs about Rituals Inventory; SSQ, Stop Signa; TAF scale, Thought-Action Fusion scale; BARI, Beliefs about Rituals Inventory; SSQ, Stop Signals Questionnaire.ls Questionnaire*.

a *Table values are given as mean ± SD unless indicated otherwise*.

b *Bold values indicate p < 0.05*.

c *Number of years spent in full-time education*.

d *Co-morbid mental disorder according to SCID and DSM-IV criteria (apart from OCD)*.

e *Treatment hours of 50 min*.

### Measures

#### OC-Specific Metacognitive Measures

The Thought-Action Fusion scale [TAF scale; (21)] has 19 items assessing thought-action fusion beliefs. It was designed to measure beliefs that having an unwanted intrusive thought increases the likelihood of specific adverse events (“Likelihood TAF”) or that it is almost the moral equivalent of carrying out that act (“Moral TAF”). The thought fusion beliefs of thought action fusion as measured by the TAF scale are found to be relevant for OCD (22). For the English version as well as the German version (23) of the TAF scale, good psychometric properties were reported. Similar to these findings, a Cronbach's alpha of 0.91 indicated a very good internal consistency in the sample of the current study.

The Beliefs about Rituals Inventory [BARI; (24)] is a 12-item questionnaire that assesses positive beliefs about rituals. For the English version, McNicol and Wells (25) reported good psychometric properties.

The Stop Signals Questionnaire [SSQ; (26)] has 12 items assessing importance of certain criteria in deciding to stop carrying out rituals. For the English version of the scale, the internal consistency was good, with a Cronbach's alpha of 0.89.

For both the BARI and the SSQ, the English versions have been translated into German by applying the back-translation technique (27), and a close match of the back-translated with the original versions has been confirmed by the authors of the BARI and the SSQ. The German versions of the BARI and SSQ showed good internal consistencies, with Cronbach's alphas of 0.85 and 0.76–0.78 in an OCD sample of a previous study (13) and in the sample of the current study, respectively.

#### Other Measures

OCD symptoms as the main treatment outcome were measured using the Yale-Brown Obsessive-Compulsive Scale [Y-BOCS; (28)], a 10-item, semi-structured interview. Y-BOCS interviews and ratings were conducted by the treating clinicians at pre-treatment, post-treatment and follow-up, interrater reliability with an independent rater was very high [*r* = 0.93–0.99, (7)]. In order to additionally measure OCD symptoms with a self-rating measure, the German Palatine Revision of the Padua Inventory [PI-PR; (29)], a 24-item questionnaire, was used. It showed a good internal consistency with a Cronbach's alpha of 0.85 in the current study.

OC-specific cognitive beliefs were assessed with the two scales “perfectionism/intolerance of uncertainty” (*perfectionism/certainty*) and “overestimation of threat/responsibility” (*threat/responsibility*) of the German version (30) of the Obsessive Beliefs Questionnaire [OBQ; (31)]. Both scales showed very good internal consistencies, with Cronbach's alphas of 0.91–0.92 in the current study. The metacognitive scale “*importance/control of thoughts*” of the OBQ was not considered in the analyses because it was not designed to measure the OC-specific metacognitions and it overlaps with them in a non-specific way.

General metacognitions were assessed using the German version (32) of the Meta-Cognitions Questionnaire [MCQ; (18)]. The MCQ showed a very good internal consistency with a Cronbach's alpha of 0.94 in the current study.

All measures were assessed at pre-treatment, post-treatment and follow-up (3 months after post-treatment).

### Statistical Analyses

Changes from pre- to post-treatment to follow-up and differences between treatment conditions in the three OC-specific metacognitions were examined by calculating separate repeated measures analyses of variances^1^ with thought action fusion, beliefs about rituals and stop signals as the dependent variables (DV). Time (pre-, post-treatment, and follow-up) and group (ERP vs. MCT) were considered as independent variables (IV) to investigate difference by treatment condition. The relevance of OC-specific metacognitions as mechanisms of change was investigated by calculating change scores for the three OC-specific metacognitions from pre- to post-treatment. To investigate these change scores in relation to alternative supposed mechanisms of change, change scores for the cognitive belief domains of “perfectionism/intolerance of uncertainty” and “overestimation of threat/responsibility” as well as general metacognitions were also calculated. In order to identify variables with relevance for the treatment outcome, correlations between the change scores and Y-BOCS and PI-PR post-treatment and follow-up scores, respectively, were calculated in a first step. In a second step, only change scores with significant correlations with treatment outcome measures were included as IV in the respective regression analyses. Moderate correlations between outcome measures (*r* = 0.29–32) justified to perform different regression analyses for the outcome variables. Four separate regression analyses were calculated for the DVs of Y-BOCS or PI-PR at post-treatment or follow-up. In all regression analyses, pre-treatment scores of the Y-BOCS or PI-PR were entered in a first step. Since the DVs did not significantly differ between treatment conditions (all *p*s > 0.428), the variable of treatment condition was not included in the regression analyses. Assumptions for these regression analyses (no multicollinearity, homoscedasticity, independent errors) were met as indicated by variance inflation factors < 1.4, the Durbin-Watson test and according histograms and P-P plots. The relevance of OC-specific metacognitions as mechanisms of change was investigated by applying four analyses leading to a high risk of familywise error rate. However, by including only predictors with significant correlations with treatment outcome and considering alternative predictors (like cognitive beliefs), these analyses are already fairly conservative. Thereby, all results are reported by applying an unadjusted alpha level of 0.05. In addition, findings, which are also significant when applying an adjusted alpha level after Bonferroni correction (α = 0.0125), are specifically highlighted.

## Results

### Change in OC-Specific Metacognitions From Pre- to Post-treatment

The effects sizes for changes in OC-specific metacognitive beliefs (as well as treatment outcomes of OCD symptoms) are displayed in Table 2. The analyses of variances revealed that both treatments led to a significant change from pre- to post-treatment in all three OC-specific metacognitions. In addition, there was a significant effect of the group x time interaction for the outcome variable of thought fusion beliefs, with the MCT treatment condition showing a stronger effect in reducing thought fusion beliefs than ERP. For beliefs about rituals and stop signals, the group x time interaction was not significant, indicating that there was no difference between treatments in reducing these OC-specific metacognitions (Table 3).

**Table 2:** Effect sizes (d_Repeated Measures_) from pre- to post-treatment (pre-post) and pre-treatment to follow-up (FU) for OC-specific metacognitions and treatment outcome of OCD symptoms.

**Outcome**		**Pre**		**Post**		**FU**		**ES**	
		***M***	***SD***	***M***	***SD***	**M**	**SD**	**Pre-post**	**Pre-FU**
**OC-specific metacognitions**									
TAF scale	ERP	1.27	0.93	1.25	1.06	1.08	0.93	0.04	0.55
	MCT	1.49	0.55	0.32	0.32	0.42	0.26	2.42	1.79
BARI	ERP	2.26	0.48	1.51	0.49	1.70	0.57	1.28	0.67
	MCT	2.55	0.67	1.38	0.40	1.30	0.29	2.91	1.91
SSQ	ERP	2.55	0.77	1.80	1.01	1.64	1.05	0.70	0.63
	MCT	2.40	0.70	1.73	1.02	1.63	0.95	0.57	0.62
**Treatment outcome of OCD symptoms**									
Y-BOCS^a^	ERP	23.82	5.81	12.55	7.81	12.55	8.01	1.46	1.63
	MCT	23.00	7.41	11.91	6.70	12.09	8.47	2.25	1.49
PI-PR^b^	ERP	1.89	0.67	0.95	0.40	0.95	0.39	1.17	1.06
	MCT	1.38	0.48	0.85	0.44	0.80	0.45	1.41	1.53

*ERP, Exposure with response prevention; MCT, Metacognitive therapy; TAF scale, Thought-Action Fusion scale; BARI, Beliefs about Rituals Inventory; SSQ, Stop Signals Questionnaire; Y-BOCS, Yale-Brown Obsessive-Compulsive Scale; PI-PR, Padua Inventory–Palatine Revision; follow-up was 3 months after posttreatment; effect size was standardized by pooled SD*.

a *Due to pairwise missings, the sample size in these analyses of variances was n = 22*.

b *Due to pairwise missings, the sample size in these analyses of variances was n = 21*.

**Table 3:** Statistics of the repeated measure analyses with the within-subject-factor Time (pre-treatment, post-treatment, and follow-up) and the between-subject-factor Group (ERP vs. MCT).

		***F***	***df***	***p^a^***	$\eta_{p}^{2}$
TAF scale	Time	23.30	18	**<0.001**	0.721
	Time × group	15.12	18	**<0.001**	0.621
BARI	Time	40.92	18	**<0.001**	0.820
	Time × group	2.19	18	0.141	0.195
SSQ	Time	3.64	18	**0.047**	0.288
	Time × group	0.02	18	0.978	0.002

*TAF scale, Thought-Action Fusion scale; BARI, Beliefs about Rituals Inventory; SSQ, Stop Signals Questionnaire. Due to pairwise missings, the sample size in the three analyses of variances was n = 21*.

a *Bold values indicate p < 0.05*.

### Prediction of Treatment Outcome by Change in OC-Specific Metacognitions

A summary of correlations is shown in the Supplementary Table 1. Regarding correlations between the change scores and the treatment outcome on the clinician-rated Y-BOCS, only changes in beliefs about rituals were significantly correlated with Y-BOCS treatment outcome at post-treatment (*r* = −0.43, *p* = 0.048) and at follow-up on trend level (*r* = −0.42, *p* = 0.053). With regard to correlations between change scores and treatment outcome on the self-rated PI-PR, changes in stop signals, general metacognitions and the cognitive belief domain “perfectionism and certainty” were significantly correlated with PI-PR post-treatment outcome (all *r*s ≥ −0.42, *p*s ≤ 0.043). In addition, only changes in stop signals significantly predicted PI-PR treatment outcome at follow-up (*r* = −0.54, *p* = 0.011).

In the regression analyses, the respective pre-treatment score was entered in step 1 of every regression analysis. Only change scores with significant correlations with the treatment outcome were included in the next steps. There was only a trend for the additional block of changes in beliefs about rituals from pre- to post-treatment in predicting Y-BOCS post-treatment outcome (Δ*r*^2^ = 0.10, *p* = 0.081). Since the correlation of changes in beliefs about rituals with Y-BOCS treatment at follow-up was close to significance, a regression analysis including BARI as IV and Y-BOCS at follow-up as DV was conducted and it also showed a trend for the additional block of the changes in beliefs about rituals in predicting Y-BOCS follow-up outcome (Δ*r*^2^ = 0.097, *p* = 0.092).

To examine whether changes in stop signals make a significant contribution over and above changes in general metacognitions and cognitive beliefs in predicting the post-treatment outcome of the self-rated PI-PR, we added changes in general metacognitions and the cognitive beliefs domain “perfectionism and certainty” in a second block and changes in stop signals in a third block. There was only a trend for the second block of changes in general metacognitions and the cognitive belief domain “perfectionism and certainty” in predicting PI-PR post-treatment outcome (Δ*r*^2^ = 0.22, *p* = 0.060) after entering pre-treatment scores of the PI-PR in step 1. Changes in stop signals of the third block explained significant additional variance of post-treatment PI-PR (Δ*r*^2^ = 0.13, *p* = 0.047) and were the only independent contributor in the last model. To examine whether changes in general metacognitions and cognitive beliefs would make a significant contribution over and above changes in stop signals, we reversed the entry of the blocks 2 and 3 of the last regression analysis. The second block of changes in stop signals explain significant additional variance of post-treatment PI-PR (Δ*r*^2^ = 0.18, *p* = 0.033), whereas there was only a trend of the third block of changes in general metacognitions and the cognitive belief domain of “perfectionism and certainty” in explaining variance of post-treatment PI-PR (Δ*r*^2^ = 0.18, *p* = 0.074). Regarding the prediction of the follow-up outcome of the PI-PR, the block of changes in stop signals explains significant additional variance (Δ*r*^2^ = 0.30, *p* = 0.011) after entering pre-treatment scores of the PI-PR in step 1. This finding is also significant by applying the adjusted alpha level after Bonferroni correction. The results of the final model for the separate regression analyses with the change scores from pre- to post-treatment as IVs and Y-BOCS and PI-PR post-treatment scores as DVs are presented in Table 4.

**Table 4:** Summary statistics for the final model of the equation in the regression of obsessive-compulsive symptoms at post-treatment and follow-up.

**Variable**	**Multiple R**	**Adj ***r***^2^**	**Beta**	***t***	**Significance^a^**
**DV: Y-BOCS post-treatment^b^**					
	0.65	0.367			
Y-BOCS pre			0.51	2.86	0.010
BARI Δ			−0.33	−1.84	0.081
**DV: Y-BOCS follow-up^b^**					
	0.64	0.351			
Y-BOCS pre			0.50	2.78	0.012
BARI Δ			−0.32	−1.77	0.092
**DV: PI-PR post-treatment**					
	0.67	0.330			
PI-PR pre			0.23	1.35	0.194
MCQ Δ			−0.19	−0.93	0.366
OBQ PC Δ			−0.31	−1.57	0.133
SSQ Δ			−0.38	−2.13	**0.047**
**DV: PI-PR follow-up^c^**					
	0.56	0.239			
PI-PR pre			0.15	0.75	0.462
SSQ Δ			−0.55	−2.82	**0.011**

*DV, Dependent variable; Y-BOCS, Yale-Brown Obsessive-Compulsive Scale; PI-PR, Padua Inventory–Palatine Revision; BARI, Beliefs about Rituals Inventory; SSQ, Stop Signals Questionnaire; MCQ, Metacognitions Questionnaire; OBQ–PC, Obsessive Beliefs Questionnaire subscale perfectionism/certainty*.

a *Bold values indicate p < 0.05*.

b *Due to pairwise missings, the sample size in this regression analysis was n = 22*.

c *Only change scores with significant correlations with treatment outcome measures were included as IV in the respective regression analyses. Due to pairwise missings, the sample size in this regression analysis was n = 21*.

## Discussion

To our knowledge, this study was the first to investigate the magnitude and impact of changes of the OC-specific metacognitions thought fusion beliefs, beliefs about rituals and stop signals after MCT and ERP treatment for OCD. The results showed that both MCT and ERP significantly reduced all three OC-specific metacognitions. Regarding thought fusion beliefs, a significant interaction effect indicated a higher decrease after MCT than ERP treatment. Changes in stop signals from pre- to post-treatment significantly predicted patient-rating OCD symptoms at post-treatment and follow-up. These changes were even predictive of treatment outcome after controlling for general metacognitions and dysfunctional cognitive beliefs. Changes in beliefs about rituals were predictive only on trend level for clinician-rating OCD symptom levels at post-treatment and follow-up.

The present study extends previous findings (15, 17) by showing that besides general metacognitions and OC-specific metacognitions (like beliefs about rituals), stop signals can be changed by treatment and that these changes are relevant for the treatment outcome in OCD. Cross-sectional studies already found that stop signals are more frequently reported by obsessional washers than non-clinical controls (14) and predict OCD symptoms after controlling for other (general and OC-specific) metacognitions and cognitions [e.g., (9, 13)]. Stop signals and beliefs about rituals both constitute the level of metacognitions in the metacognitive model of OCD, which guide the response to negative appraisals of thoughts. Being directly related to rituals, they might constitute a vulnerability factor for falling back into previous behavioral responses in critical situations and might thereby be important to change by treatment. This would also explain why changes in these metacognitions in the current study do not only predict treatment outcome at post-treatment but also at follow-up 3 months later.

Not only MCT but also ERP seems to be able to reduce these OC-specific metacognitions. Beliefs about rituals and stop signals might not have to be explicitly discussed during treatment as it is applied in MCT. The experience of going through exposures without using rituals might question positive beliefs about the necessity to perform rituals. Patients might replace subjective with more objective criteria for stopping behaviors when they learn to go without rituals during exposures. However, more treatment time was needed to achieve changes in these metacognitions using ERP. MCT seems to be more time-efficient by directly targeting these OC-specific metacognitions, and this efficient change might also be advantageous for attaining a similar reduction of OCD symptoms as in ERP in less treatment time [cf., (7)].

In line with previous studies (15, 17), our findings suggest that a change in OC-specific metacognitions might be more important for the treatment outcome than a change in dysfunctional cognitive beliefs. Accordingly, treatments should additionally or alternatively focus on thought processes and not necessarily thought contents, like it is implemented in the cognitive therapy. However, changes in the cognitive belief domain “perfectionism and certainty” were predictive on trend level for treatment outcome in the present study. In addition, a prediction of post-treatment outcome was not significant after applying the adjusted alpha level with Bonferroni correction. Future research is necessary to disentangle a possible interplay between a change of metacognitions and a change of cognitions, which are only considered as by-products of metacognitions in MCT (1). Our findings additionally indicate that targeting OC-specific rather than general metacognitions is beneficial for treatment outcome in OCD.

Although MCT was advantageous in changing thought fusion beliefs, this change did not predict treatment outcome in the current study. Findings of cross-sectional studies already suggest that thought fusion beliefs might not be OC-specific but pronounced in different clinical groups [e.g., (33)], compared with non-clinical groups [e.g., (13)]. The metacognitive model states that the activation of fusion beliefs only leads to negative appraisals of the thought as dangerous, which further result in OCD symptoms through the activation of beliefs about rituals and stop signals. Thereby, fusion beliefs might not necessarily cause OCD symptoms and their reduction might not necessarily result in a better treatment outcome in OCD. However, since Grotte et al. (15) found a prediction by fusion beliefs, future studies should further investigate their predictive value and increased changeability by MCT in the treatment of OCD.

One of the study's advantages is that the changeability of OC-specific metacognitions was investigated in a trial comparing MCT with ERP. MCT for OCD specifically aims to change these metacognitions, whereas ERP is supposed to work on other mechanisms of change. The high treatment fidelity we found in the pilot trial comparing ERP and MCT by Glombiewski et al. (7) suggested that MCT-specific components were delivered according to protocol without contamination. The current study could thereby shed first light on an improved effect of MCT in reducing OC-specific metacognitions. Another advantage of the study is the investigation of all OC-specific metacognitions, which allowed for an integrated view of their impact on the treatment outcome. The additional consideration of general metacognitions and cognitive beliefs enables us to evaluate the predictive meaning of different dysfunctional beliefs being related to different clinical implications (e.g., focusing on metacognitive vs. cognitive beliefs during treatment).

One major limitation is the small sample size. In addition, there might be further variables that are predictive of treatment outcome but have not been considered in the present study. For instance, the therapeutic relationship has been discussed as an important general factor for the outcome in psychotherapy. Especially in a treatment setting in which patients have to go through difficult situations (like exposures or behavior experiments), the relationship between the patient and therapist might be a crucial factor for the treatment success. A strong therapeutic alliance might also improve patient adherence (e.g., with regard to homework compliance) which was found to be a robust predictor of ERP treatment outcome in OCD [for a review, see Wheaton and Chen (34)]. However, the present study aimed to focus on factors that are relevant according to specific treatments like MCT and cognitive therapy.

The present study has clinical implications suggesting that ERP and MCT might share metacognitive mechanisms of change. As one of them, stop signals were partly predictive of OCD treatment outcome. MCT treatment techniques like directly challenging beliefs about rituals and stop signals by using verbal methods and behavioral experiments can lead to a time-efficient reduction of these metacognitions and subsequently of OCD symptoms. In addition, the present findings suggest placing an increased focus on metacognitions dealing with the behavioral response than on metacognitions about the importance of thoughts. It seems to be beneficial to work on processes of thoughts instead of thought contents in the treatment of OCD. The present study findings justify a larger trial with a focus on the mechanisms of metacognitions, their relevance for the treatment outcome and the treatment elements affecting them.

## Data Availability Statement

The raw data supporting the conclusions of this article will be made available by the authors, without undue reservation.

## Ethics Statement

The studies involving human participants were reviewed and approved by Institutional Review Boards of the University of Marburg and of the University of Leipzig. The patients/participants provided their written informed consent to participate in this study.

## Author Contributions

CE and JG initiated the study. CE, JG, and JH designed the study and wrote the study proposal. JH analyzed the data and drafted the manuscript. CE, JG, and AH revised the manuscript. All authors helped in collecting the data, contributed to the interpretation of data, critically reviewed for important intellectual contents, and gave the final approval of the version to be published.

## Conflict of Interest

The authors declare that the research was conducted in the absence of any commercial or financial relationships that could be construed as a potential conflict of interest.

## Publisher's Note

All claims expressed in this article are solely those of the authors and do not necessarily represent those of their affiliated organizations, or those of the publisher, the editors and the reviewers. Any product that may be evaluated in this article, or claim that may be made by its manufacturer, is not guaranteed or endorsed by the publisher.

## Footnotes

^1^Multilevel models using maximum likelihood estimation, with a fixed intercept, an indicator variable for condition, an indicator variable for time and a condition x time interaction term showed similar results as these ANOVAs with a significant effect of time for all OC-specific metacognitions and a significant condition × time interaction term for the variable of thought action fusion.
