# Supplementary material for: Metacognitive Change During Exposure and Metacognitive Therapy in Obsessive-Compulsive Disorder
Source: Front Psychiatry. 2021 Sep 3;12:722782. doi: 10.3389/fpsyt.2021.722782 (PMC8446266; doi:10.3389/fpsyt.2021.722782)
Supplement: Supplementary file 1 [file Table_1.DOCX]

**Table S1**

|  |  | 1 | 2 | 3 | 4 | 5 | 6 | 7 | 8 | 9 | 10 |
| --- | --- | --- | --- | --- | --- | --- | --- | --- | --- | --- | --- |
| 1 | Y-BOCS post-treatment^a^ |  | **.85*** | .32 | .30 | .23 | **-.43*** | -.22 | -.11 | .23 | .16 |
| 2 | Y-BOCS follow-up^a^ |  |  | .29 | .30 | .16 | -.42 | -.18 | -.18 | .20 | .34 |
| 3 | PI-PR post-treatment |  |  |  | **.67*** | .03 | -.19 | **-.42*** | **-.47*** | **-.42*** | -.04 |
| 4 | PI-PR follow-up^a^ |  |  |  |  | -.05 | -.23 | **-.54*** | -.18 | -.13 | -.05 |
| 5 | TAF scale Δ |  |  |  |  |  | .26 | .04 | .37 | .33 | .12 |
| 6 | BARI Δ |  |  |  |  |  |  | .35 | **.56*** | .15 | .08 |
| 7 | SSQ Δ |  |  |  |  |  |  |  | .25 | -.02 | -.04 |
| 8 | MCQ Δ |  |  |  |  |  |  |  |  | **.46*** | .24 |
| 9 | OBQ - PC Δ |  |  |  |  |  |  |  |  |  | **.56*** |
| 10 | OBQ - TR Δ |  |  |  |  |  |  |  |  |  |  |

*Intercorrelations for changes in belief domains (metacognitive and non-metacognitive) and obsessive-compulsive symptoms.*

Y-BOCS: Yale-Brown Obsessive-Compulsive Scale; PI-PR: Padua Inventory - Palatine Revision; TAF scale: Thought-Action Fusion scale; BARI: Beliefs about Rituals Inventory; SSQ: Stop Signals Questionnaire; MCQ = Metacognitions Questionnaire; OBQ - PC = Obsessive Beliefs Questionnaire subscale perfectionism/certainty; OBQ - TR = Obsessive Beliefs Questionnaire subscale threat/responsibility.

*Correlations are significant at *p* < .05. ^a^Due to missings, samples sizes are n = 22 (Y-BOCS posttreatment and follow-up), n = 21 (PI-PR follow-up).
